# Lymphedema pathogenesis involves antigen-driven expansion of CD4+ T cells in skin

**DOI:** 10.3389/fimmu.2025.1620571

**Published:** 2025-08-01

**Authors:** Adana-Christine Campbell, Annica R. Stull-Lane, Jung Eun Baik, Ananta Sarker, Jinyeon Shin, Gopika Ashokan, Hyeung Ju Park, Bracha L. Pollack, Pradhi Pakkerakari, Yollanda Franco Parisotto, Arielle Roberts, Chrysothemis C. Brown, Babak J. Mehrara, Raghu P. Kataru

**Affiliations:** ^1^ Plastic and Reconstructive Surgery Service, Department of Surgery, Memorial Sloan Kettering Cancer Center, New York, NY, United States; ^2^ Immuno-Oncology Service, Human Oncology and Pathogenesis Program, Memorial Sloan Kettering Cancer Center, New York, NY, United States

**Keywords:** lymphedema, T cells, antigen-specific responses, insulin, oligoclonality

## Abstract

**Introduction:**

Lymphedema, a progressive condition involving unresolved swelling and inflammation, affects as many as 1 in 1000 Americans. Although CD4+ T cells are implicated in the chronic inflammatory process, antigen-specific responses are understudied.

**Methods:**

Using high-throughput sequencing, we studied the T cell receptors (TCRs) of CD4+ T cells in paired normal and lymphedema skin biopsies of 11 patients. We also employed *in vitro* studies using human samples and cells from a lymphedema mouse model.

**Results:**

Target epitopes of the TCRs, including the antigen insulin, were identified. Clonality was significantly higher in lymphedema samples than in controls, both in human samples and a mouse model of the disease. *In vitro* studies using human samples and a lymphedema mouse model demonstrated increased activated memory T cell responses specific to the antigen insulin compared with the control.

**Discussion:**

Our study highlights an oligoclonal expansion of CD4+ T cells in lymphedema and supports insulin as a probable antigen driving T cell responses. These findings can help inform more precise therapeutic targets for the development of better therapies and preventative tools to combat lymphedema progression.

## Graphical Abstract

Clonal expansion of T cells in lymphedema: a graphical abstract. Illustration depicting the clonal expansion of T cells in lymphedema, divided into four stages. Stage 1 shows leaky lymphatics allowing fluid leakage. Stage 2 displays antigens in fluid presented by antigen-presenting cells. Stage 3 illustrates the oligoclonal CD4+ T cell response. Stage 4 highlights the release of proinflammatory cytokines promoting inflammation. Each stage is visually represented with corresponding cells and fluid flow. This image was created in https://BioRender.com.

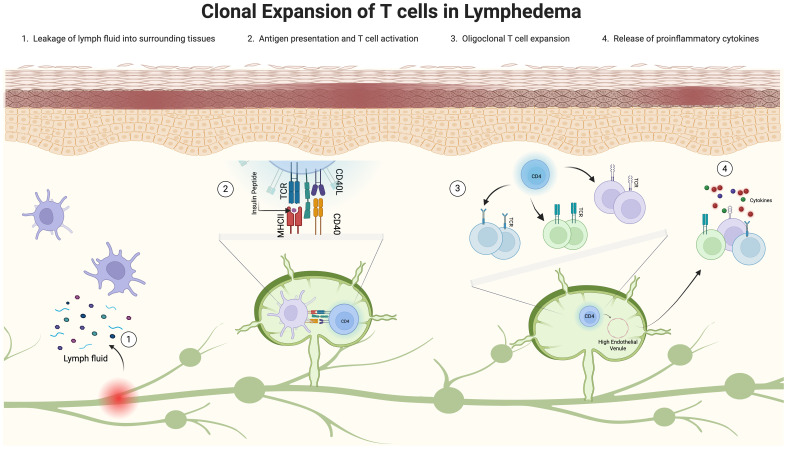

## 1 Introduction

Secondary lymphedema is a chronic and progressive disease characterized by swelling, fibrosis, and recurrent infections. Over 5 million Americans are affected by lymphedema (1); however, it is unclear why some patients develop more severe lymphedema than others. There is a paucity of effective treatments for all patients. Although conservative therapies provide symptomatic relief, they do not prevent disease progression, and surgical treatments are limited (1, 2).

The importance of T cells in the pathogenesis of lymphedema is well-established, and lymphedema has been recognized as a T-cell-mediated disorder (3, 4). In particular, the CD4+ T cell lineage has been shown to comprise most T cells in lymphedema clinical and murine tissues (5, 6). Sustained lymphatic stasis results in CD4+ T cell inflammation, leading to fibrosis and inflammatory lymphangiogenesis (3, 5). Studies in mouse models demonstrate that depletion of CD4+ T cells prevents lymphedema development (7, 8). The number of infiltrating CD4+ T cells is significantly correlated with the degree of inflammation and severity of lymphedema (4, 6, 7), and T helper type 2 (Th2) differentiation is necessary for the histopathological changes observed in lymphedema. Together, these prior works underscore the critical role of CD4+ T cells in the chronic inflammatory process of lymphedema.

Although evidence implicates CD4+ T cells in site-specific inflammation and lymphatic dysfunction, the immunologic drive responsible for the accumulation of T cells in sites of lymphatic stasis remains unknown. CD4+ T cell activation requires an interaction with antigen-bearing dendritic cells (9), a process that can also occur in lymphedema (4). As such, T cell activation in lymphedema may occur because of antigens present in tissues with lymphatic fluid stasis. Clonotypic analyses of infiltrating T cells in other Th2-mediated inflammatory diseases provide evidence for antigen-driven accumulation of T cells (10–12). The T cells in skin lesions in atopic dermatitis are oligoclonal, indicating involvement of antigen-specific immune reactions (10, 11, 13). Oligoclonal expansion of intradermal T cells has also been demonstrated in psoriatic skin lesions (12, 14). The detection of antigen-specific T cells in disease states has been challenging because of the relatively low frequencies of CD4+ T cells sequenced from tissue and the multitude of potential target epitopes that can exist for a given sample (15). This limitation has improved in recent years with the generation of peptide libraries that allow for the predictive epitope binding of T cells analyzed by immunosequencing techniques.

In this study, we hypothesized that T cell immune responses in lymphedema are antigen-specific and oligoclonal. We aimed to validate this by analyzing T cell receptor (TCR) sequencing in lymphedematous tissues to identify putative antigens driving clonal T cell expansions in lymphedema. The clinical significance of this study lies in the identification of a common clonal architecture among patients with lymphedema, thereby facilitating early clinical detection of at-risk patients. Employing biopsy samples from patients with lymphedema, we demonstrated that the CD4+ T cell response is an oligoclonal, antigen-driven expansion. A notable antigen identified was autologous insulin. Our findings were further substantiated by *in vitro* studies demonstrating selective T cell activation in lymphedema in response to insulin. Together, these findings suggest that future lymphedema treatment approaches should focus on identifying antigenic stimuli or elimination of pathogenic T cell clones.

## 2 Materials and methods

### 2.1 Human subjects

Following Institutional Review Board approval, samples were collected from 11 adult female patients (**Table 1**) diagnosed with unilateral breast cancer-related lymphedema and treated at the Lymphedema Clinic in the Plastic and Reconstructive Surgery Service at Memorial Sloan Kettering Cancer Center in New York, NY, USA. Additional patient characteristics are included in **Supplementary File 1**.

**Table 1:** Clinical data of study patients.

Patient	Age (years)	BMI (kg/m2)	Race	ISL	Duration of disease (months)	Volume differential (%)	L-Dex	Radiation (Y/N)
1	52	26.85	W	2	132	43.87	57.5	Y
2	58	24.4	W	2	102	21	20.1	Y
3	51	23.7	W	2	30	32	45.9	Y
4	54	27.8	W	2	15	42	52.7	Y
5	60	28.24	W	2	240	52.18	49.9	N
6	63	26.7	W	2	3	19	21.3	Y
7	54	19.4	W	2	122	24.4	17.5	Y
8	52	26	UNK	2	84	39.8	38.7	N
9	68	29.43	W	2	157	33.3	71.6	Y
10	53	23.63	W	2	101	61.34	71.9	N
11	56	21.9	W	2	19	17.5	43.4	N

BMI, body mass index; UNK, unknown.

### 2.2 Animals

All animal procedures were performed in accordance with the protocol approved by the Institutional Animal Care and Use Committee at Memorial Sloan Kettering Cancer Center. Female C57BL/6J (wild type) mice were purchased from the Jackson Laboratories and experiments were started at age 8–10 weeks. All mice were maintained in a pathogen-free, temperature- and light-controlled environment and provided with a normal chow diet and freshwater.

### 2.3 DNA extraction, TCRβ high-throughput sequencing, and analysis

#### 2.3.1 Human samples

For TCR sequencing, genomic DNA was isolated from punch biopsies taken from the affected and non-affected limbs using a QIAamp DNA formalin-fixed and paraffin-embedded (FFPE) tissue kit (Qiagen, cat#56404). TCRβ regions were sequenced from a standardized quantity of quality-controlled genomic DNA using an immunoSEQ analyzer (Adaptive Biotechnologies). In brief, the assay uses a 2-step bias-controlled multiplex PCR system that spans the TCRβ VDJ regions at lengths specific to the CDR3 region of each T cell clone. Deep sequencing techniques were used to generate a unique TCR library for each sample. The immunoSEQ assay outputs a file that includes the percentage of T cells in a sample, the relative frequency of top clones, CDR3 length, amino acid sequence, and V/J gene usage between samples. All reads were normalized to allow cross-sample comparisons of repertoire clusters. Data analysis was performed using the immunoSEQ analyzer 3.0 software.

#### 2.3.2 Animal samples

We used the previously described mouse tail model of lymphedema in animals in the experimental group (n=5) (16). Briefly, the superficial and deep lymphatic vessels were ligated through a 2-mm circumferential excision of the tail skin 20-mm far from the base of the tail. For control group (n=5) animals underwent sham surgery where a superficial incision is made without removing the skin or dermal lymphatics. One-centimeter longitudinal tail sections centered on the wound as well as cross sections located 1 cm proximal/distal to the wound were harvested at 6 weeks post procedure and fixed overnight in 4% paraformaldehyde. Tissues were decalcified, embedded in paraffin, and sectioned into 10-µm units. Genomic DNA was extracted using a QIAamp DNA FFPE tissue kit (Qiagen, #56404). Immunosequencing of the TCRβ VDJ regions was performed using an immunoSEQ analyzer (Adaptive Biotechnologies), as described above. Data analysis was performed using the immunoSEQ analyzer 3.0 software.

### 2.4 Determination of TCRβ epitope sequences

The corresponding epitope sequence recognized by each unique T cell clone sequenced in the lymphedema samples was determined using a validated online TCR structural repertoire database (17). The database contains a curated collection of TCR structures from the Protein Data Bank and ranks the corresponding epitope sequence of each TCR based on the PAM30 scoring matrix. The PAM30 index is a bioinformatics metric that matches protein sequences based on the lowest number of amino acid substitutions. The lower the PAM30 index, the greater the similarity in sequence alignment. Using the amino acid construct of the TCR generated by the immunoSEQ™ analyzer for the top 5 unique clones detected in each lymphedema sample, the corresponding epitope sequence was determined using the TCR3d database.

### 2.5 Predictive antigen analysis

The NCBI BLAST was used to search and identify the predictive antigen corresponding to each unique TCR sequenced in the lymphedema samples. BLAST identifies regions of similarity between biological sequences (18, 19). The program compares nucleotide or protein sequences to sequences in a curated database and organizes the queried amino acid sequence pairs based on the best alignment. The search was limited to human organisms (Homosapien sapiens). BLAST then generates a description of the predicted antigen based on matched sequences, producing significant alignments. It then ranks the antigen based on the percent identity (the maximum and total alignment score for the database sequence), the query coverage (the percentage of the query sequence that is covered by the database sequence alignment), and the expect value (E-value). The E-value is the number of expected hits with similar scores that could be found by chance. The lower the E-value, the more likely the query-pair alignment is significant.

### 2.6 Histological analysis

Histology was conducted as described above. In brief, human tissue samples were embedded in paraffin and sectioned into 5-µm sections. The following anti-human antibodies were used for staining: CD4+ (1:1000; cat#AF379NA; R&D Systems), CD45RO (1:400; cat#MA511532; Thermo Fisher Scientific), and IR (1:1000; cat#AB137747; Abcam). Tissues were rested overnight with the appropriate primary antibody at 4°C, washed with PBS, and incubated with the corresponding secondary antibody conjugates (TRITC, Cy3, and FITC) at room temperature for 5 hours. A 4,6-diamino-2-phenmylindole (DAPI; cat# D4571, Molecular Probes) stain was performed to identify nucleated cells. Sections were scanned using a Mirax slide scanner (Zeiss), and ImageJ software (National Institutes of Health) was used to quantify CD4+/CD45RO+ and CD4+/CD45RO+/IR+ cells per DAPI+ cells in a 20-µM area.

### 2.7 Cell sorting, *in vitro* cell culture, and flow cytometric analysis

#### 2.7.1 Human samples

Heparinized whole blood and liposuction fluid samples were obtained from the same donor and processed on the same day. Peripheral blood mononuclear cells (PBMCs) were isolated from blood by density-gradient centrifugation using Ficoll-Paque Plus (#17144002; Cytiva). Any residual red blood cells (RBCs) from samples were lysed with RBC lysis buffer (#00-4333-57; Invitrogen). Lipoaspirate samples and PBMCs were then processed with a CD4+ T cell negative selection kit (#130-096-533; Miltenyi Biotec). T cells were prepared in R10 media and seeded at 50–100,000 cells per well in a 96-well plate and cultured with co-stimulatory molecules CD49d and CD28, with either PepTivator Insulin (#130-096-771; Miltenyi Biotec) peptide pool stimulation, B 9–23 peptide (AS-61532; Anaspec Inc.), whole insulin (I5523; Sigma-Aldrich) or vehicle control, in the presence or absence of anti-insulin receptor antibody S961 (#S6922; Selleckchem.com). Wells were stained with anti-CD4, anti-CD45, anti-CD45RO, anti-CCR7, anti-CD44, anti-CD154, and IR and then analyzed by flow cytometry.

#### 2.7.2 Animal samples

A PLND model was used as previously described (20, 21). Briefly, the hindlimb collecting vessels and popliteal LNs were identified and the LNs were excised with the popliteal fat pad. For control group animals underwent sham surgery where a superficial incision is made without LN excision. At 2 weeks, surgical mice and sham controls (n=7 per group) were euthanized by CO_2_ inhalation, and the ipsilateral, draining, inguinal LN was collected. Single-cell suspensions were prepared from inguinal nodes pooled from each group and sort purified for effector TCRβ^+^CD4^+^CD44^hi^CD62L^lo^ cells after enrichment with a CD4^+^ T cell negative isolation kit (cat#130-104-454; Miltenyi Biotec). Similarly, antigen-presenting cells (APCs) were isolated from single-cell suspensions from one spleen harvested from a sham control and irradiated after depletion of T cells using antibodies against CD90.2 (cat#14-0902-82; Invitrogen).

Irradiated APCs (2 x10^4^ cells/well) were plated in a 96-well U-bottom plate and pulsed with 100 ug/mL of 1 mg insulin B (10–24) peptide (cat#AS-61532; Anaspec) in triplicate at 37°C for 2 hours or left unstimulated. Plates were washed with PBS and then co-cultured with sorted effector T cells from either sham controls or surgical mice at a 1:5 ratio in 200 µL of media and rested at 37°C for 48 hours. At 4 hours before harvest, wells were treated with brefeldin A (cat#420601; Biolegend), washed with PBS, stained with anti-CD4, anti-TCRβ, anti-CD44, anti-CD62-L, and anti-CD 154 (for activated effector T cell populations), and analyzed by flow cytometry.

### 2.8 Statistical analysis

Statistical analysis was performed using GraphPad Prism v8 (GraphPad Software). We used a two-tailed paired t-test to compare normally distributed paired data and a two-tailed unpaired t test to compare normally distributed unpaired data. Nonparametric testing with Wilcoxon signed-ranked test was used for data that were not normally distributed. Significance was defined as P<0.05.

## 3 Results

### 3.1 T cell repertoires in lymphedema skin demonstrate oligoclonal expansion

**Figure 1:**
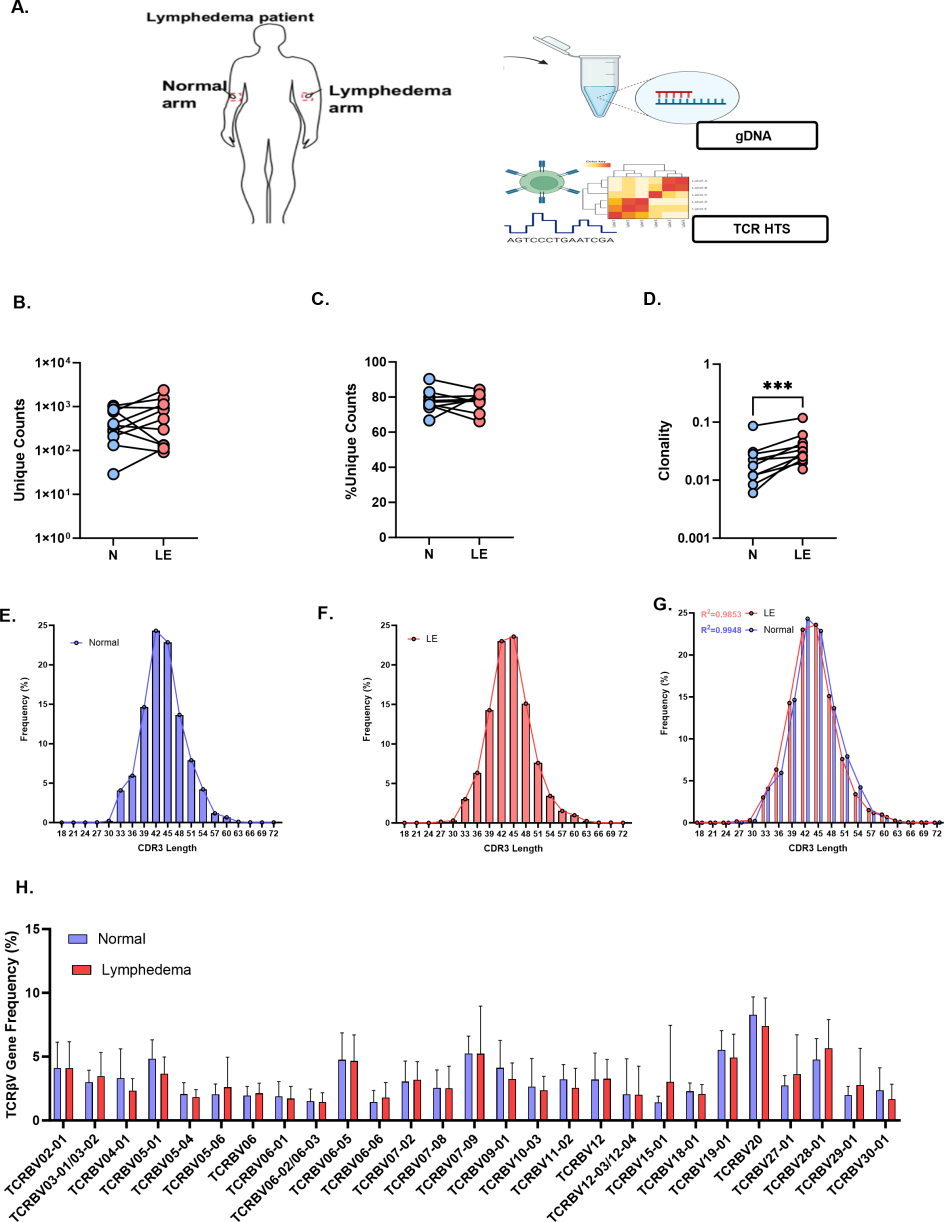
TCR repertoire analysis of CD4+ T cells in paired lymphedema and normal skin biopsies. **(A)** Schematic of collection and sequencing of CD4+ T cells in human skin samples (n=11), genomic(g) DNA isolated and high-throughput sequencing (HTS) performed using 2-step bias-controlled PCR. **(B-D)** Unique counts, percent unique counts, and clonality index of TCRs in normal and lymphedema (LE) samples. **(E)** CDR3 AA length distribution in normal skin. **(F)** CDR3 AA length distribution in LE skin. **(G)** Overlap of CDR3 length usage in normal and LE skin. **(H)** TCRβV gene usage in normal and LE skin. Student’s paired t-test; ***p<0.0001.

Patients with secondary lymphedema were recruited from the Lymphedema Clinic in the Plastic and Reconstructive Surgery Service at Memorial Sloan Kettering Cancer Center (**Table 1** **,** **Supplementary File 1** **).** For TCR sequencing, genomic DNA was isolated from full-thickness punch biopsies of normal and lymphedematous skin in patients with International Society of Lymphology (ISL) stage II lymphedema (**Figure 1A**). TCR sequencing of skin samples revealed 1.0–1.5 thousand unique sequences per paired sample, comprising the majority of clones sequenced (**Figures 1B, C**). The Simpson clonality index was used to measure the diversity of the T cell repertoires in a sample (**Figure 1D**). Measures closer to 0 indicate a perfectly diverse repertoire or no duplication of clones, whereas measures closer to 1 indicate a monoclonal population with one clone dominating a repertoire (Adaptive Biotechnologies). Oligoclonal populations fall within this range and are recognized as repertoires with significantly expanded clones, which may indicate a response to putative antigens.

In our study, T cells sequenced in lymphedema skin samples demonstrated an increased degree of oligoclonal expansion compared with clones detected in matched normal skin samples (**Figure 1D**, p<0.0001). CDR3 length distribution is an additional method used to determine repertoire diversity. The average CDR3 lengths in this study did not differ significantly between normal and lymphedema samples. Additionally, both groups were observed to fit well within a Gaussian distribution curve (R^2^≈1; **Figures 1E–G**). Variable (V) gene recombination was also identified in subsets of sequenced T cells. V gene usage appeared to be conserved between TCRs in normal and lymphedema samples. Among the 30 high-frequency expressed V genes (frequency >1.0%), the greatest variation was observed in TCRβV-20, although this was not found to be significant between normal and lymphedema samples (**Figure 1H**).

### 3.2 Clonally propagated CD4+ T cells in lymphedema are patient-specific

**Figure 2:**
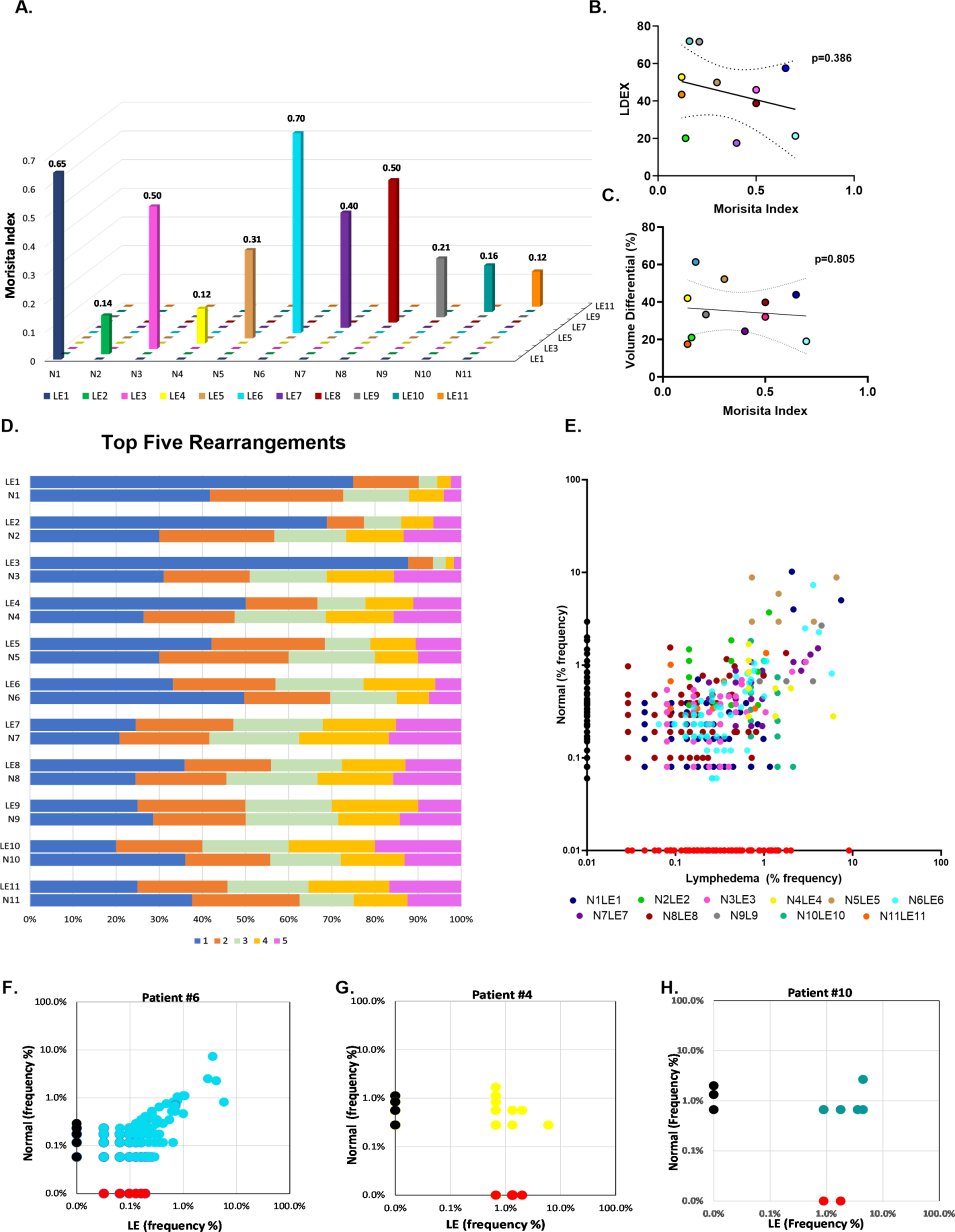
Comparative analysis of TCR profiles in normal and lymphedema skin biopsies. **(A)** Comparison of similarity of TCR repertoires between paired normal and lymphedema (LE) samples according to the Morisita overlap index. Individual patients defined by colored bars; closer to zero indicates more dissimilar repertoires and closer to 1 indicates more similar repertoires. **(B, C)** Correlation of high and low Morisita indices with L-Dex and volume differential. **(D)** Top 5 clonally propagated T cells in paired normal and LE samples; (x-axis) frequency percent clonal expansion relative to other top clones in the sample. **(E)** Clonal expansion of shared T cell clones. Colored dots indicate individual patient. **(F-H)** Exemplary patient samples demonstrating expansion of shared clones in LE skin.

The Morisita overlap index (MOI) was used to assess the similarity of TCR repertoires between paired normal and lymphedema skin samples, as well as among patients with the disease (**Figure 2A**). The MOI ranges from zero to one; the closer the index is to one, the more similar the repertoires, and the closer the index is to zero, the more dissimilar the repertoires. Some patients demonstrated high TCR similarity between normal and lymphedema skin (MOI>0.5; patients 1 and 6), whereas some patients had very dissimilar repertoires (MOI<0.2; patients 2, 4, 5, 9, 10 and 11). No overlap was observed among patients in our cohort, suggesting that TCR repertoires in lymphedema are patient-specific. We then compared the MOI to L-Dex (ImpediMed), a lymphedema measuring system that compares fluid levels in the affected and non-affected limb (22), and volume differential (**Figures 2B, C**) and found no significant correlation with TCR similarity (p>0.05).

To better delineate clone distribution within a repertoire, we identified the frequencies of each donor’s top 5 clones, which were significantly expanded in the sample relative to other clones (**Figure 2D**). Notably, clones are defined by their unique amino acid rearrangements. Single clone expansion among top clones was commonly observed in lymphedema skin (>50% frequency, x-axis), whereas top clones in normal skin tended to have a more even distribution, arguing for a strong T cell response to certain antigens in lymphedema skin. A pair-wise scatter plot was similarly used to visualize the relative abundance of shared clones between normal and lymphedema samples (**Figure 2E**). Clone frequency in normal tissue alone was plotted on the y-axis, clone frequency in lymphedema tissue alone was plotted on the x-axis, and the clone frequency in both normal and lymphedema tissues were displayed as individually colored dots. We observed that lymphedema skin had the highest frequency of shared clones, suggesting that T cells encounter antigens in lymphedema skin that are absent in the contralateral normal limb (**Figures 2F–H**).

### 3.3 Predictive antigen binding of top clones in lymphedema demonstrates increased insulin affinity

Specific TCR target antigens were predicted for top clones sequenced from lymphedema skin using a two-stage immunoinformatics approach. Briefly, the amino acid identity of the TCR was identified through immunosequencing, and a list of antigenic major histocompatibility complex-2 (MHC-II) epitopes was generated using a validated, online TCR structural database. Corresponding MHC epitopes were ranked by PAM30 score, with a lower score indicating fewer amino acid rearrangements of the input sequence or best match. A PAM30 score of less than 90 was used to determine the most accurate MHC-II antigen-TCR pair.

To search and identify candidate antigens for sequenced TCRs, we used the National Center for Biotechnology Information (NCBI) Basic Local Alignment Search Tool (BLAST). **Table 2** summarizes the representative antigens and the number of unique TCRs recognizing those antigens. Details on this search are provided in the methods section. Interestingly, human insulin was the most common antigen detected by frequently propagated clones in lymphedema and was identified by unique TCRs in 10 of the 11 samples sequenced. Other representative antigens, detected at lower frequencies, included Klebsiella bacterial antigen, gluten plant antigen, and the HIV viral antigen. **Supplementary File 2** lists several parameters for this analysis, including predictive antigen, percent identity, and E-value. We chose to move forward with investigaing the role of insulin, since it was the most representative antigen detected of the patient cohort.

**Table 2:** Summary of TCR antigen specificity in lymphedema skin using BLAST analysis.

Antigen Parameters	Bacterial Antigen	Self Antigen	Plant Antigen	Viral Antigen
Unique TCRs mapped to antigen (*n*)	6	15	2	2
Representative antigen	Klebsiella pneumoniae	Insulin	Gluten	HIV/ Herpesvirus
Lymphedema patient samples with TCRs that recognize antigen (*n*)	3	10	3	1
Description	Klebsiella pneumoniae	Human insulin and insulin analogs	Bread wheat	HIV gag polyprotein, EBV viral protein

### 3.4 CD4+ T cells in lymphedema exhibit an activated memory phenotype response to insulin antigen

**Figure 3:**
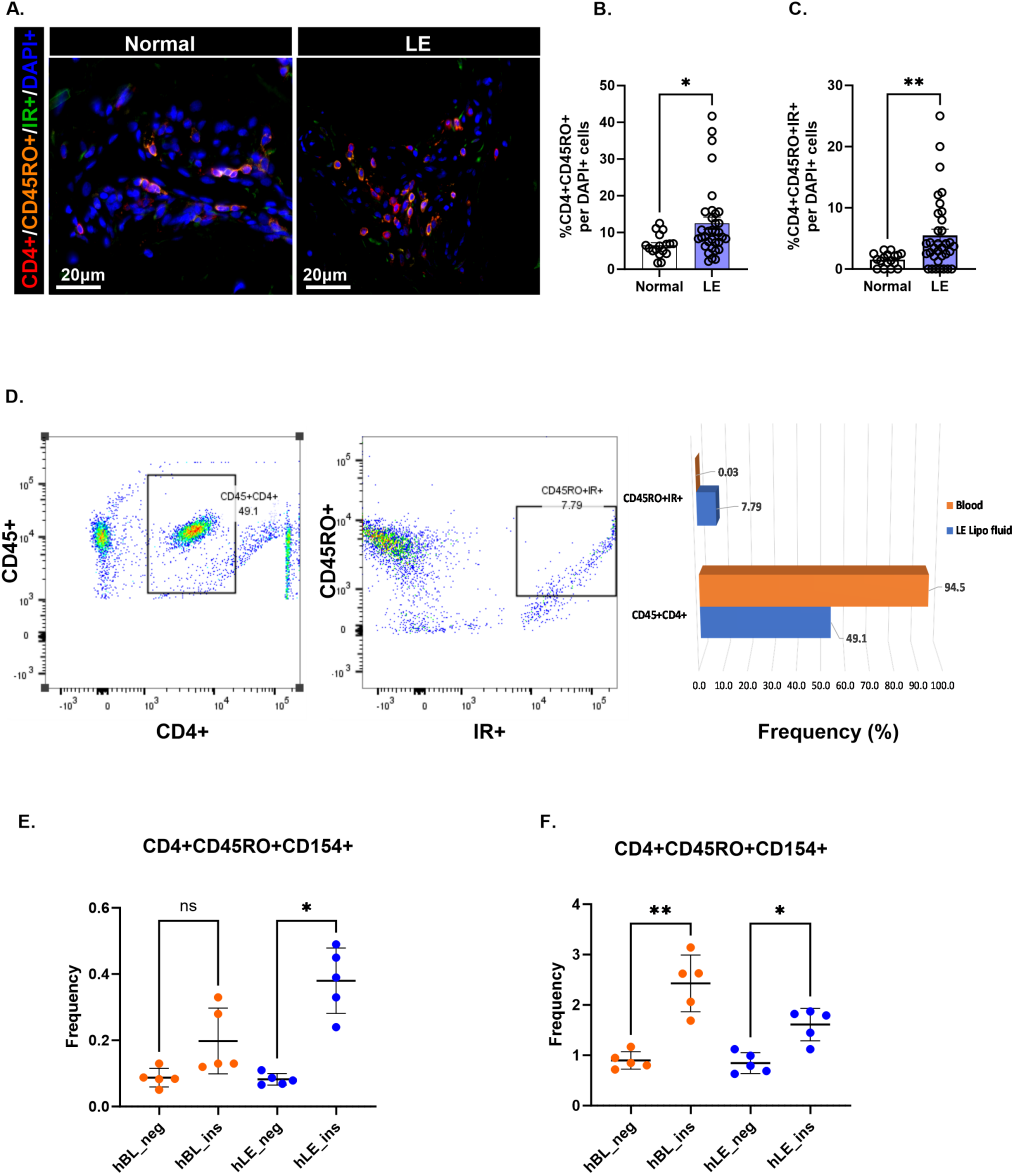
CD4+ T cells in lymphedema exhibit an effector memory phenotype response to insulin peptide. **(A)** Immunofluorescence images showing effector memory CD4+ T cells (CD4+CD45RO+) and IR-activated effector memory CD4+ T cells (CD4+CD45RO+IR+) in normal and lymphedema (LE) skin biopsies. **(B, C)** Quantification **(D)** Gating strategy and frequency percent of antigen-activated (CD45RO+IR+) CD4+ T cell populations in LE liposuction fluid. A full gating strategy for panel **(D)** can be viewed in **Supplementary File 3**. **(E, F)** Frequency (%) insulin responsive T cell populations between human LE fluid T cells (hLE) and human blood T cells (hBL) in two single human donors with LE and BMI of <25 **(E)** and >25 **(F)**. The full gating strategy for panels **(E, F)** may be viewed in **Supplementary File 4**. Data analyzed by One-way ANOVA. *p<0.05, **p<0.005; ns, not significant.

Immunofluorescence staining provided further evidence of significantly higher number of antigen-responsive T cells, as illustrated by the accumulation of activated T cells (CD4+/CD45RO+) in lymphedema skin compared with matched normal skin (**Figures 3A, B**). A significant number of these activated T cells are IR+ in lymphedema skin compared with matched normal skin (**Figure 3C**). Similarly, we observed higher frequencies of antigen-activated CD4+ T cell populations in lymphedema liposuction fluid (CD4+CD45RO) than in autologous blood on flow cytometry, supporting a local T cell response to antigens in lymphedema that is absent systemically (**Figure 3D**; **Supplementary File 3**). CD4+ T cells isolated from lymphedema liposuction fluid stimulated *ex vivo* with a peptide pool from human insulin also demonstrated increased frequencies of antigen-activated T cells (CD4+CD45RO+CD154+) compared with autologous T cells from blood in a patient with normal BMI (**Figure 3E**; *p<0.05). A second patient with an overweight BMI demonstrated reactive T cells both systemically and in lymphedematous tissue (**Figure 3F**; *p<0.05, **p<0.005; **Supplementary File 4**).

### 3.5 Oligoclonality and antigen-activated effector CD4+ T cells are demonstrated in mouse lymphedema models

**Figure 4:**
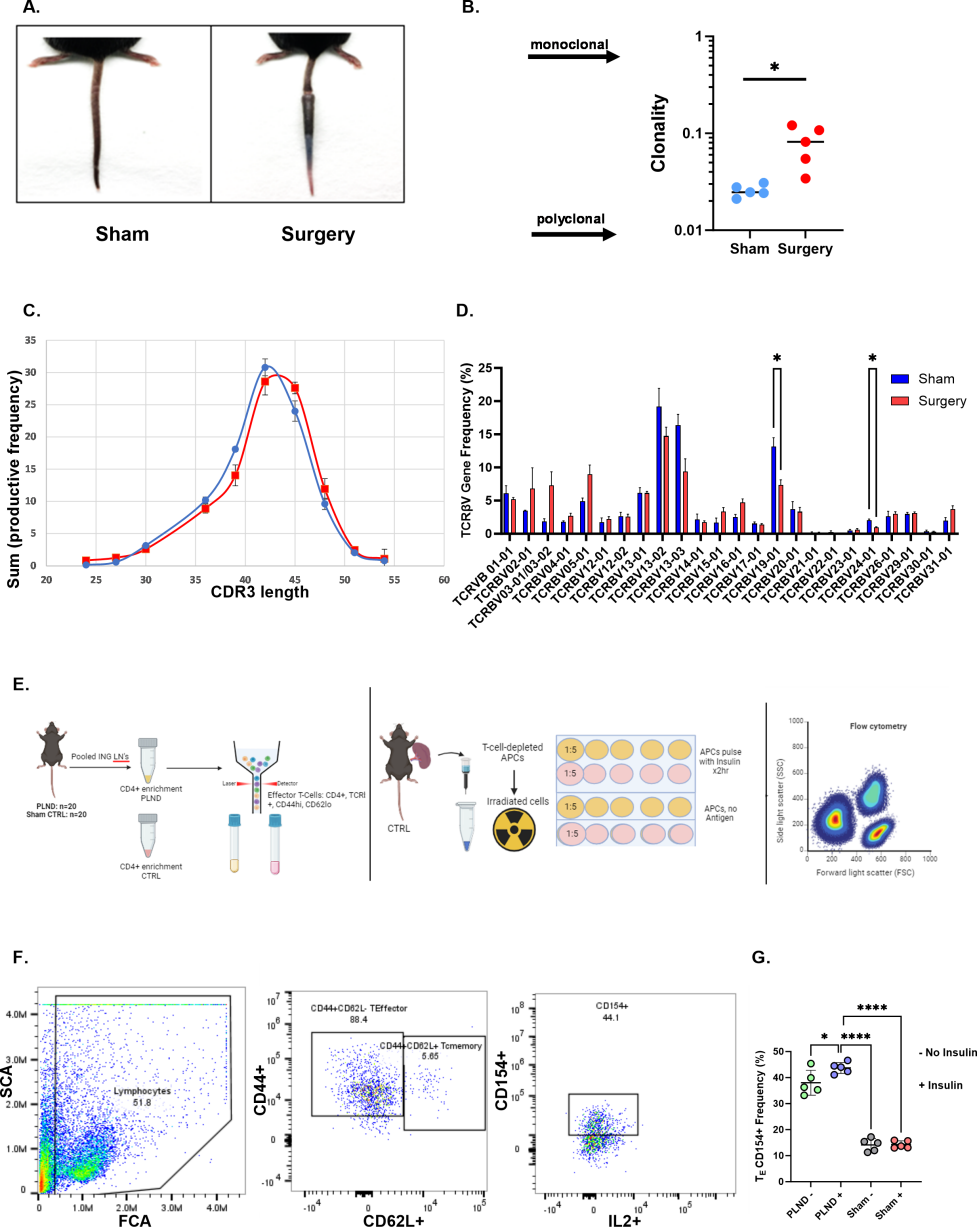
Oligoclonality is demonstrated in a lymphedema mouse model. **(A)** Representative tail images of sham and tail-operated mice at 6 weeks. **(B)** Clonality index of CD4+ TCRs sequenced in tail skin of sham and surgery mice. A two-tailed unpaired t-test was performed. **(C)** CDR3 AA length distribution in sham (blue) and surgery (red) mice. **(D)** TCRβV gene usage in sham and surgery mice. Data analyzed by a two-tailed multiple unpaired t-test. **(E)** Experimental schematic for mouse studies: Effector T cells are sorted from PLND and sham controls (left panel). Irradiated APCs are plated with effector T cells at a 1:5 ratio, with or without insulin antigen, and treated for 48 hrs (middle). Samples are analyzed by flow cytometry for antigen-activated effector populations. **(F)** Gating strategy of populations of interest. The full gating strategy for panel **(F)** may be viewed in **Supplementary File 5**. **(G)** Comparison of effector T cell populations expressing CD154. Data analyzed by one-way ANOVA. *p<0.05; ****p<0.0001.

T cell oligoclonality, as determined by the Simpson clonality index, was similarly demonstrated in a tail lymphatic excision mouse model of lymphedema (**Figures 4A, B**). CDR3 length distribution exhibited a skewed pattern in the surgical group compared with the sham control, suggesting increased oligoclonality (**Figure 4C**). Comparison of TCRβV gene usage between the sham and surgery groups demonstrated significantly increased gene usage of TCRβV 19–01 and TCRβV 24–01 in the sham group (**Figure 4D**; p=0.05). Antigen-activated effector CD4+ T cells were examined in a popliteal lymph node dissection (PLND) model of lymphedema. Two factors influenced our decision to use the PLND model for this experiment. First, T cell yield from tail skin and associated draining lymph node (LN) sites (sacral nodes) was consistently low in our repeat experiments. Second, the PLND model improves T cell yield from draining inguinal LNs with increased cell viability, as it eliminates the need for additional digestion steps required in the tail skin model. **Figure 4E** illustrates our experimental design. In brief, CD4+/CD44+/CD62L- effector T cells isolated from draining inguinal LNs of the PLND model were stimulated *in vitro* with or without insulin. Effector T cells isolated from the PLND group demonstrated significant upregulation of CD154+, an antigen-specific marker, compared with effector cells either in the absence of insulin or effector cells stimulated with insulin in sham controls (**Figures 4F, G**; **Supplementary File 5**).

### 3.6 Human T cell responses in lymphedematous tissue are related to insulin peptide and whole insulin

**Figure 5:**
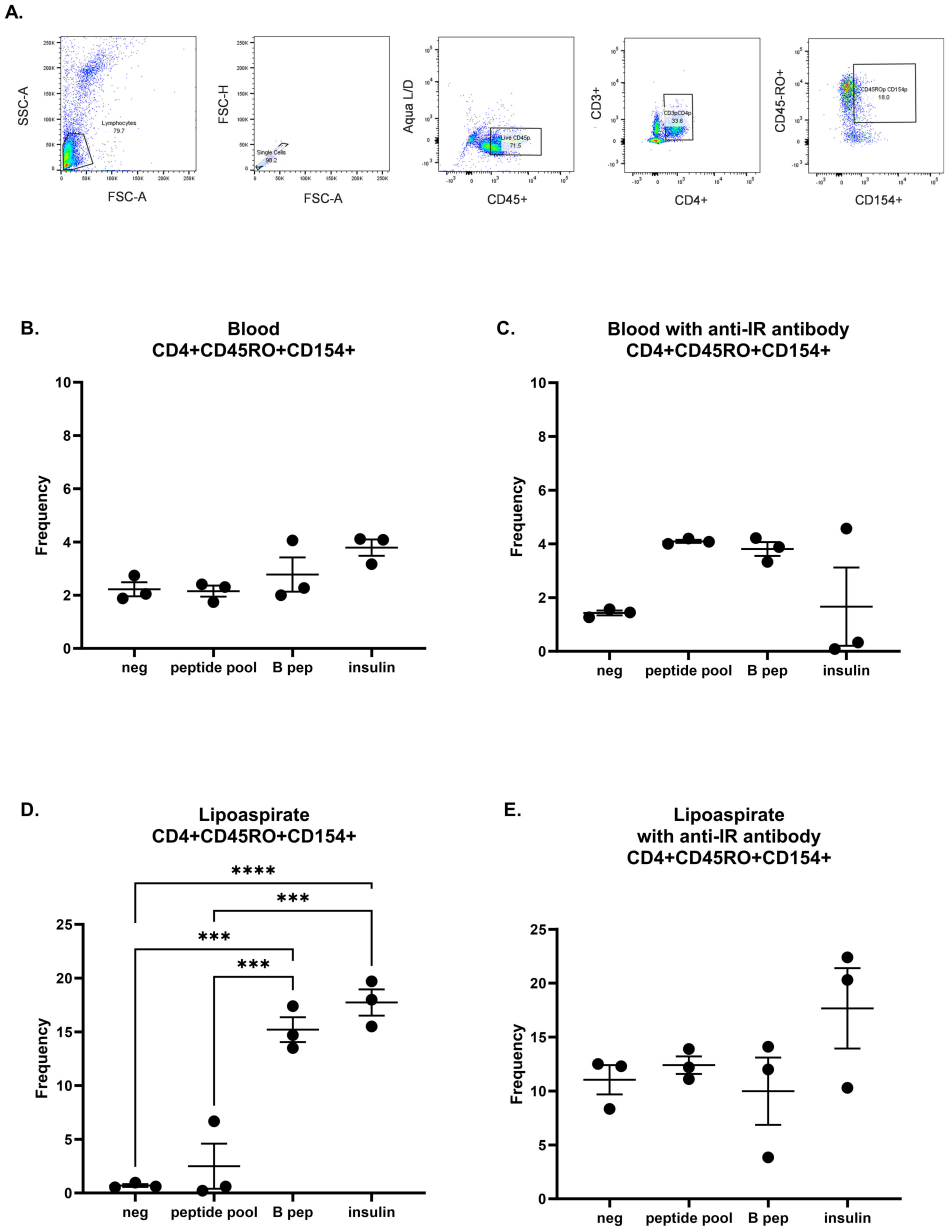
Human T cell responses in lymphedematous tissue are specific to insulin peptide and whole insulin. **(A)** Gating strategy for human activated memory T cells. T cells were isolated from human blood **(B, C)** and autologous lipoaspirate **(D, E)** and incubated with negative control or insulin stimulation (peptide pool, B 9–23 peptide, and whole insulin) in the presence or absence of anti-insulin receptor antibody. Each graph was analyzed with a one-way ANOVA and Tukey’s multiple comparisons test; ***p<0.001; ****p<0.0001. All unmarked comparisons were not significant.

To test additional sources of insulin and the specificity of insulin as antigen, we designed an experiment to compare T cells from blood and T cells from lymphedematous tissue (lipoaspirate), incubated in the presence or absence of anti-insulin receptor (IR) antibody. We assessed the samples in triplicate per condition with flow cytometry and gated for activated memory T cells (**Figure 5A**). No significant differences were present between groups in stimulated T cells isolated from blood, either incubated without or with anti-IR antibody (**Figures 5B, C**). T cells from lipoaspirate from the same patient were found to be significantly responsive to B 9–23 peptide and whole insulin (**Figure 5D**; p<0.05), whereas these differences were no longer significant when incubated in the presence of anti-IR antibody (**Figure 5E**). These results suggest that T cells may be activated through both insulin receptor binding as well as via binding by insulin peptide-loaded MHC class II molecules. Taken together, a graphical abstract (**Graphical Abstract**) is provided.

## 4 Discussion

Our results support that antigen recognition plays an important role in T cell expansion in lymphedema and underscore the hypothesis that specific antigens drive these responses. Human insulin was identified as the most common antigen detected by lymphedema-associated T cells. Further, we assessed patient samples and found that memory T cells isolated from lymphedema fluid responded to stimulation with human insulin peptides *ex vivo* with a pronounced increase in insulin receptor expression, whereas autologous T cells from blood did not. These results suggest an autoimmune component in CD4+ T-cell-driven lymphedema pathology, with human insulin as the autoantigen.

Autoimmunity has been extensively studied in the pathogenesis of type 1 diabetes (23), and insulin has been described as a major autoantigen, especially insulin B-chain peptide (B9-23) (24–26). Insulin acts on target cells via the insulin receptor (IR), which can be differentially expressed depending on the target cell and local environment (27). Whereas the most studied target cells are skeletal muscle, liver, and fat (28), immune cells are also affected by local insulin concentrations. Interestingly, a type 1 diabetes study in mice demonstrated that T cells with a high expression of IR were aggressively diabetogenic, and a follow-up study determined that the pathogenic cells with high IR expression are predominantly memory CD4+ T cells (29, 30).

The roles of insulin and inflammation have also been extensively studied in obesity and type 2 diabetes (31). Adipose tissue-residing macrophages, dendritic cells, and adipocytes can all play a role in antigen presentation to T cells (32). Lymphatic dysfunction secondary to diet-induced obesity involves chronic inflammation, such as peri-lymphatic accumulation of macrophages and CD4+ T cells, and the consequences of hyperglycemia and insulin resistance, including decreased pumping capacity and increased lymphatic leakiness (33–35).

Metabolic dysfunction has been studied in lymphedema, both in relation to obesity and in relation to the lymphedema microenvironment. BMI has a direct correlation with development of lymphedema (35, 36). Obesity induces expression of adipokines and free fatty acid accumulation, which in turn compromises lymphatic function (35). With respect to the lymphedema microenvironment, independent of obesity, lymphatic injury can lead to excess fluid accumulation and alterations of the metabolism of lymphatic endothelial cells (37). Specifically, mitochondrial respiration, the most efficient mechanism for generating energy in the cell, is reduced in lymphatic endothelial cells in lymphedema, which could lead to a decrease in lymphangiogenesis and an exacerbation of lymphedema. Together, during lymphedema pathogenesis, the local adipose deposition and lymphatic insufficiency can cause insulin resistance and an inclination towards metabolic syndrome (35).

Both obesity and lymphedema involve a chronic inflammatory process, and T cells have been implicated (35–38). Interestingly, we found that a patient with increased BMI had T cell responses both systemically and in lymphedematous tissue, whereas a patient with normal BMI had responses only specific to the lymphedematous tissue. While human samples are more heterogenous than mouse, this observation is important in understanding contributions of underlying metabolic disease. It is possible that metabolic syndrome increases the amount of circulating insulin-reactive T cell clones, which in turn would exacerbate lymphedema pathology.

Insulin has been described as an antigen in diabetes and non-diabetic conditions. In type 1 diabetes, insulin-reactive CD4+ T cells have been implicated in autoimmune pathophysiology (39). These insulin-specific T cells invade islets and destroy insulin-secreting beta cells (40). Insulin autoantibodies may also be detectable in insulin-naïve individuals that have autoimmune disorders (41). None of the 11 patients assessed had a diagnosis of diabetes, suggesting that the local insulin-specific responses seen were related to lymphedema. How might a self-protein like insulin become antigenic in lymphedema? At homeostasis, many self-antigens are carried by the lymph and are involved in immune tolerance (42), whereas lymphedema involves lymphatic fluid stasis and an abnormal buildup of protein-rich fluid (43). Accumulated lymph with high levels of insulin may contribute to impaired immune tolerance (44) and/or a buildup of oxidative neoantigen forms of insulin with altered post-translational modifications due to the inflammatory microenvironment in lymphedematous tissues (45). While more research is needed to elucidate this concept fully, it is clear that lymphedema has many similarities to other Th2 diseases and autoimmune conditions (4, 8, 46).

Local hyperinsulinemia in lymphedematous tissues has been described in prior studies (27, 47). The pathophysiology of lymphedema secondary to lymphatic injury involves lymphatic fluid stasis, lipid accumulation, and elevated insulin levels in lymph fluid (43). A previous study reported insulin levels in lymph fluid from lymphedema patients to be more than 20-fold higher than insulin levels in plasma; the authors concluded that insulin likely acts as an adipogenic factor in lymphedema pathogenesis, contributing to the proliferation and differentiation of adipose-derived stem cells (48). Our study builds upon this by describing that T cells isolated from lipoaspirate are activated by insulin, whereas autologous T cells isolated from peripheral blood are not, in a patient with normal BMI. Patient and clinical characteristics play a role, as T cells from the blood of a patient with an overweight BMI were activated by insulin in addition to the lipoaspirate T cells. More studies are needed to tease apart the role of pre-existing metabolic syndrome and obesity in the insulin-activated T cell mechanism of lymphedema. In some cases, insulin-activated T cells may contribute to early disease, since previous studies have identified CD4+ T cells in general as important mediators of lymphedema (5, 6). However, depending on the patient with respect to cancer therapy, comorbidities, among other factors, whether insulin-reactive T cell clones are a cause or consequence of lymphedema is yet to be elucidated. Further, the detected clones may be recruited and expanded, or may be *in situ* generated, or it may be a combination of both. Future studies may tease apart these nuances. 

Overall, while there may be mechanistic similarities between the pathogeneses of diet-induced and/or autoimmune diabetes and lymphedema pathophysiology, questions remain unanswered and effective pharmacologic prevention or treatment of lymphedema is still lacking. Metformin has shown a positive response in reducing lymphedema pathology (49, 50). Researchers have also suggested that glucagon-like peptide-1 (GLP-1) receptor antagonist treatment may also decrease the risk of developing lymphedema (47, 51). More research into therapeutics that target insulin resistance may help in the treatment of lymphedema. Given the heterogeneity of CD4+ T cell populations and their ability to regulate local metabolic status in adipose tissues (31), future studies should focus on additional immunophenotyping of T cell subpopulations in lymphedema patients whose symptoms resolve as compared to lymphedema patients whose symptoms progress to characterize protective immunophenotypes. Further, future studies could focus on patient-specific TCR repertoires for potential personalized treatment approaches.

This study has some limitations. While a strength is the comparison of lymphedematous skin and the contralateral unaffected side from the same patients, future studies could include additional samples from age-matched healthy controls as well as breast cancer patients without lymphedema. This study focused on breast cancer-related lymphedema, since it is the most common cause of secondary lymphedema in the United States, and it represents a unique population seen at MSKCC. Worldwide, it is estimate that 3 to 5 million patients are affected by breast cancer-related lymphedema (52). Future studies would need to investigate the generalizability of the role of insulin-activated T cells in primary lymphedemas, which present earlier in life with a genetic etiology, or other secondary lymphedemas such as in relation to the parasitic disease filariasis (53). Further, our sample size of 11, although meaningful for some bioinformatic analyses, is a limitation in terms of generalizability. Nonetheless, other high-impact studies have similarly included the paired breast cancer-related lymphedema samples from lymphedema and non-lymphedema arms and analysed with an N less than 11, depending on the bioinformatic analysis utilized (54).

## Data Availability

The raw data supporting the conclusions of this article will be made available by the authors upon request, without undue reservation.
