# Supplementary material for: Lymphedema pathogenesis involves antigen-driven expansion of CD4+ T cells in skin
Source: Front Immunol. 2025 Aug 1;16:1620571. doi: 10.3389/fimmu.2025.1620571 (PMC12354532; doi:10.3389/fimmu.2025.1620571)
Supplement: Supplementary file 1 [file Presentation1.pptx]

## Slide 1
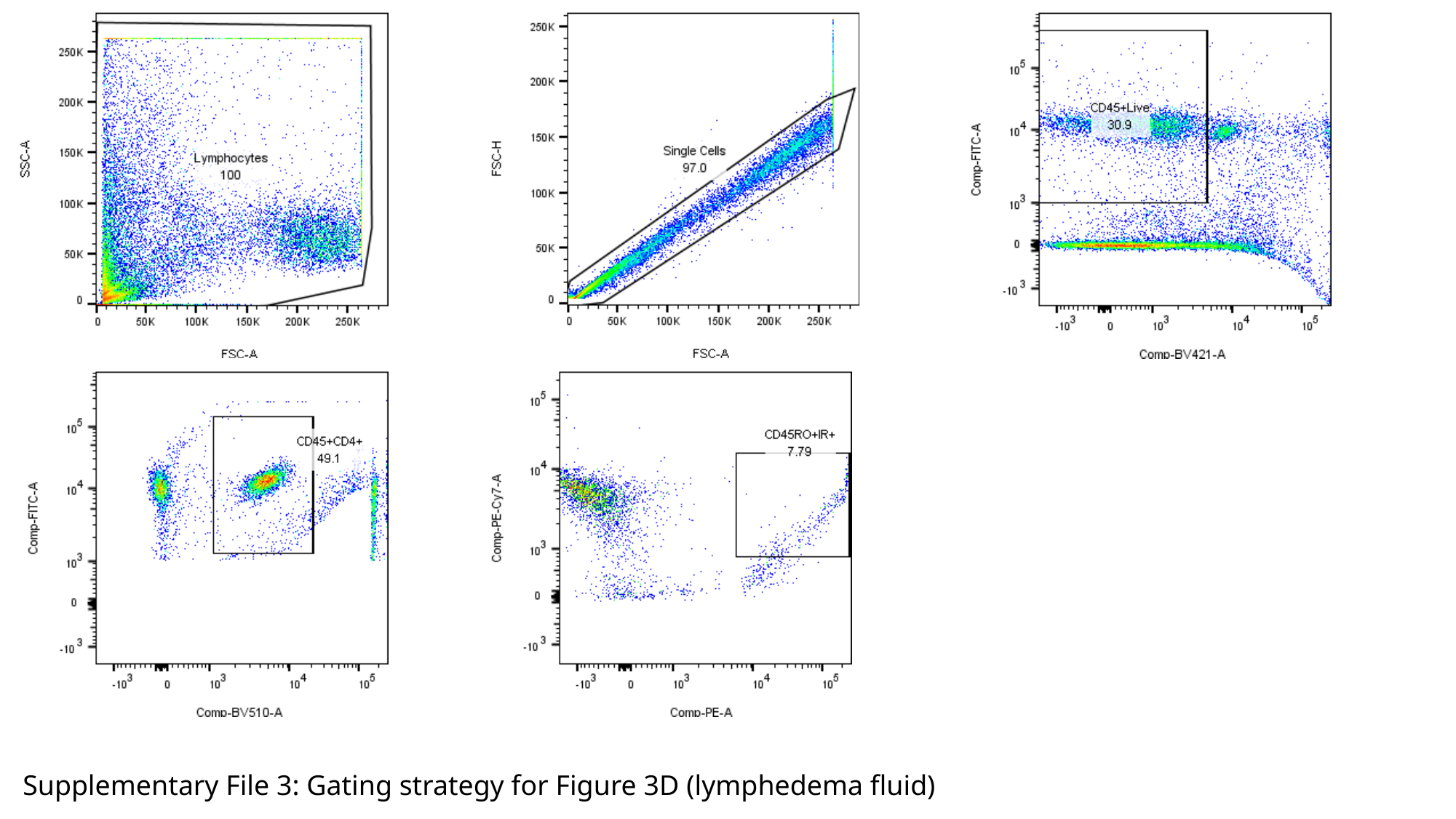

Supplementary File 3: Gating strategy for Figure 3D (lymphedema fluid)

## Slide 2
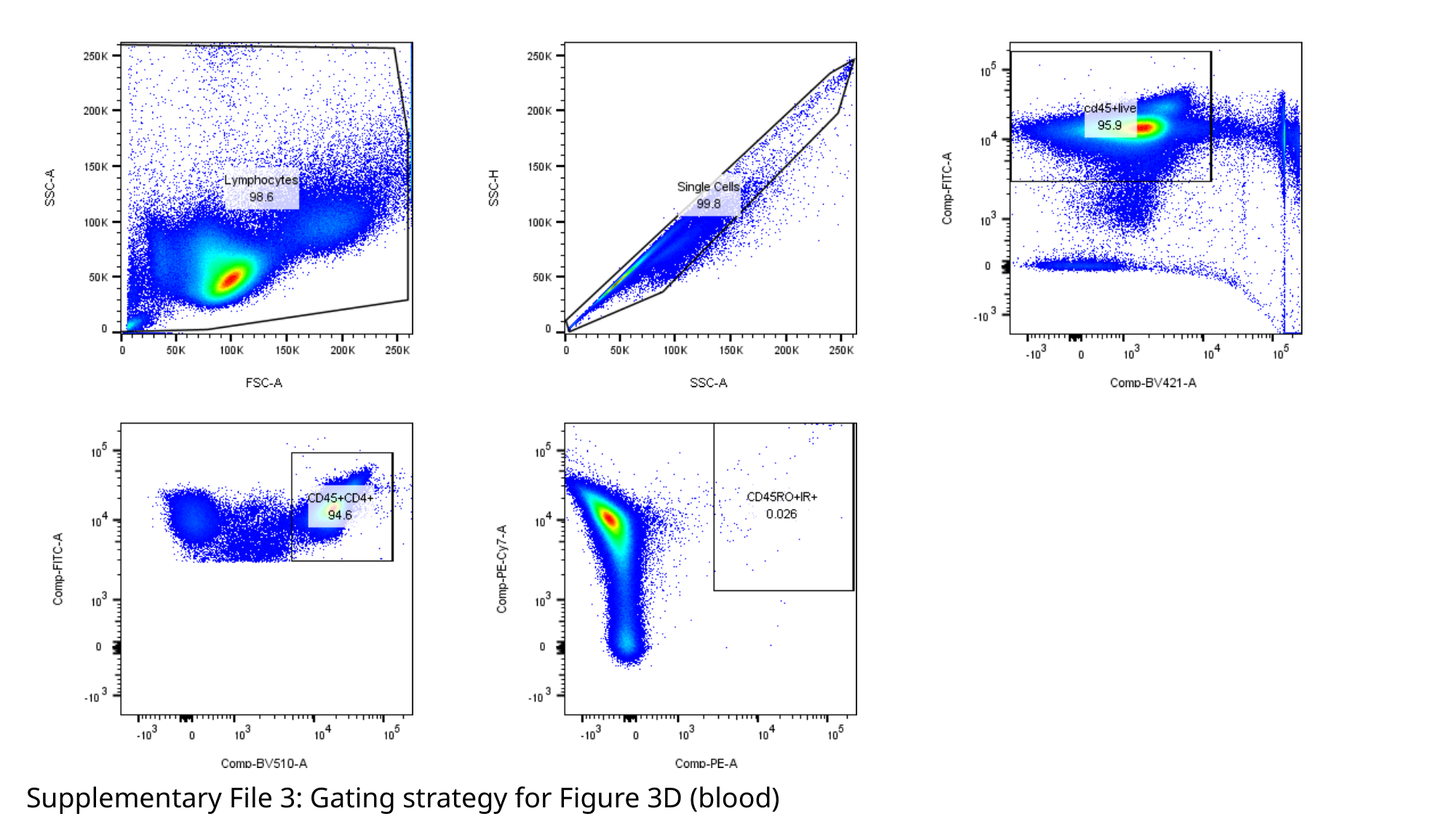

Supplementary File 3: Gating strategy for Figure 3D (blood)
