# Supplementary material for: Lymphedema pathogenesis involves antigen-driven expansion of CD4+ T cells in skin
Source: Front Immunol. 2025 Aug 1;16:1620571. doi: 10.3389/fimmu.2025.1620571 (PMC12354532; doi:10.3389/fimmu.2025.1620571)
Supplement: Supplementary file 2 [file Presentation2.pptx]

## Slide 1
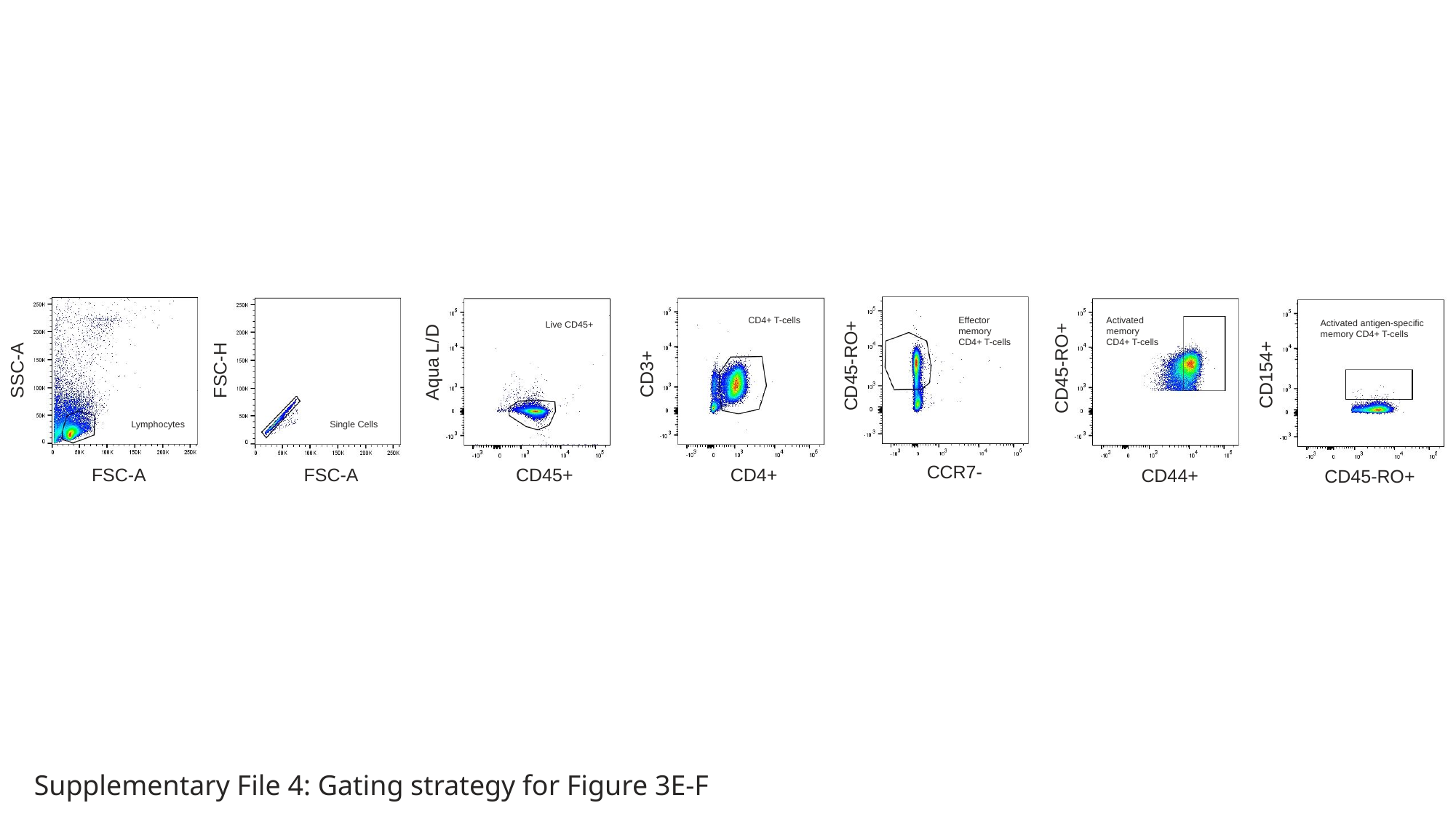

CD4+ T-cells
Effector memory CD4+ T-cells
Activated memory CD4+ T-cells
Activated antigen-specific memory CD4+ T-cells
Live CD45+
Aqua L/D
CD45-RO+
CD45-RO+
SSC-A
FSC-H
CD3+
CD154+
Lymphocytes
Single Cells
CCR7-
FSC-A
FSC-A
CD45+
CD4+
CD44+
CD45-RO+
Supplementary File 4: Gating strategy for Figure 3E-F
