# Supplementary figures and images for: Lymphedema pathogenesis involves antigen-driven expansion of CD4+ T cells in skin
Source: Front Immunol. 2025 Aug 1;16:1620571. doi: 10.3389/fimmu.2025.1620571 (PMC12354532; doi:10.3389/fimmu.2025.1620571)

## Slide 1
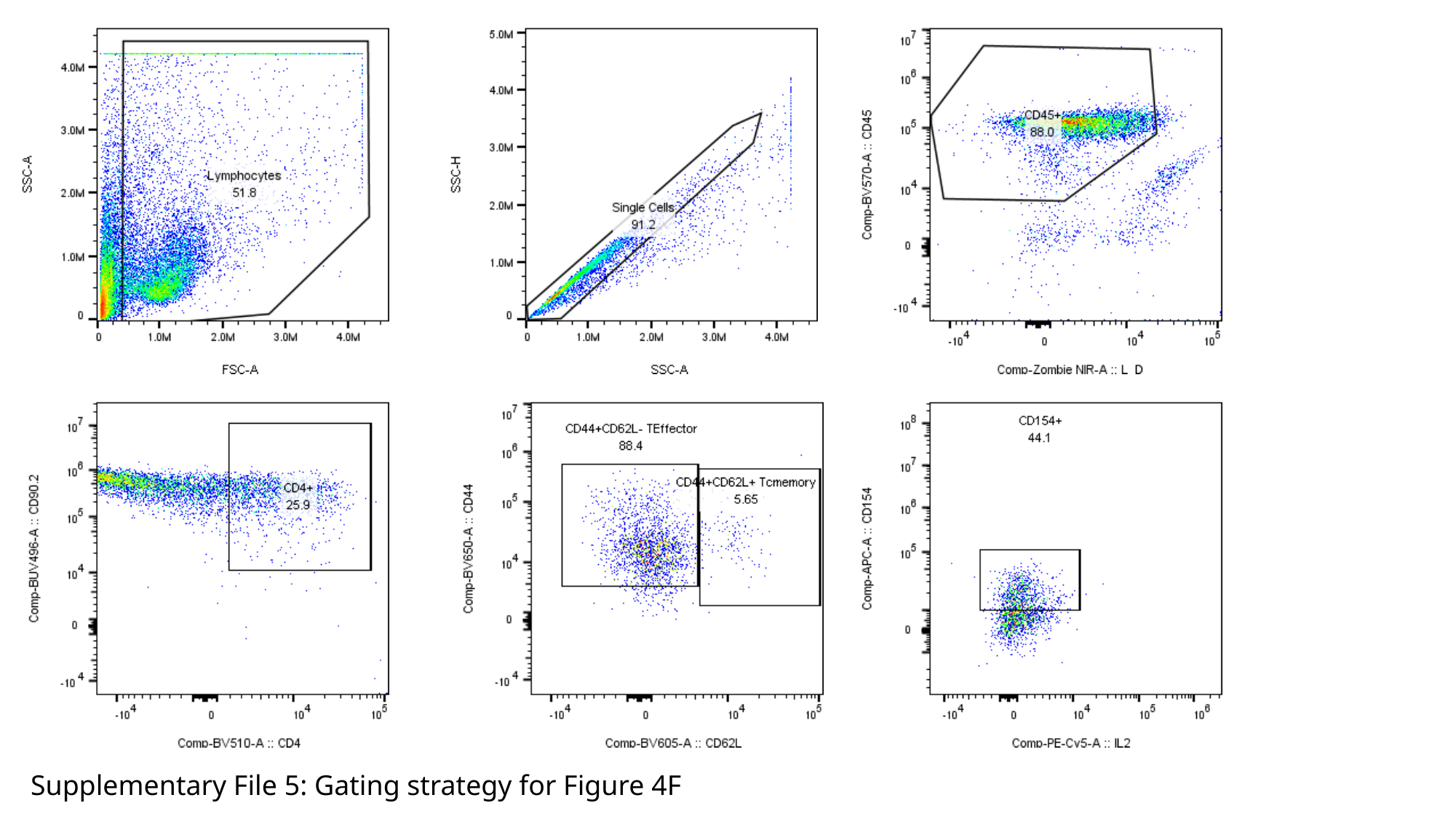

Supplementary File 5: Gating strategy for Figure 4F

Supplement: Supplementary file 3 [file Presentation3.pptx]
